# Length of Stay, Readmission Rates, and Mortality Are Similar Between Hospitalized Individuals With Sickle Cell Disease With and Without COVID-19

**DOI:** 10.7759/cureus.70567

**Published:** 2024-09-30

**Authors:** Karen A Clarke, Mohamad Moussa, Mary Ann Kirkconnell Hall, Yoo Mee Shin

**Affiliations:** 1 Division of Hospital Medicine, Emory University School of Medicine, Atlanta, USA

**Keywords:** clinical outcomes, covid-19, length of stay, mortality, sickle cell disease

## Abstract

Background

We sought to clarify the impact of COVID-19 on clinical outcomes in sickle cell disease patients, given their baseline hypercoagulable state in combination with COVID-19-related coagulopathies and other complications.

Methods

Retrospective chart review of two groups of sickle cell disease patients hospitalized between March 2020 to December 2021: Group 1 did not have COVID-19 (n = 95) and Group 2 did (n = 73).

Results

Groups 1 and 2 were similar in terms of age, race, sex, comorbid illnesses, genotype, hydroxyurea use, and opioid use. Group 1 and 2 patients had a mean hospital length of stay of 7.05 and 7.64 days, respectively (p = 0.981). ICU-level care was required for six (6.3%) Group 1 patients and four (5.5%) Group 2 patients (p = 1.000). Readmissions within 30 days occurred for 25 (26.3%) Group 1 patients, and 18 (24.7%) Group 2 patients (p = 0.807). Death occurred for one (1.05%) Group 1 patient and one (1.4%) Group 2 patient (p = 1.000). There were no significant differences in commonly ordered initial laboratory values (total bilirubin, hemoglobin, hematocrit, creatinine, lactate dehydrogenase, and D-dimer) between Group 1 and Group 2 patients.

Conclusions

We observed no significant differences in clinical outcomes among sickle cell disease patients hospitalized due to COVID-19 compared to those without COVID-19.

## Introduction

Since the severe acute respiratory syndrome coronavirus 2 (SARS-CoV-2) virus was first identified in December 2019, more than 772 million people have acquired this viral infection, and more than 6.9 million of these have died due to COVID-19, the disease caused by this virus [1]. Elderly patients and those who have pre-existing illnesses are at increased risk for developing severe complications and experiencing increased mortality due to COVID-19 [2].

Sickle cell disease is the most commonly occurring hemoglobinopathy worldwide [3]. It is caused by a point mutation in the β-globin gene, which leads to multiple acute and chronic severe complications that can affect almost every organ in the body [4,5]. Life expectancy for patients with sickle cell disease is 30 years less at their usual baseline compared with the general population [4]. Patients with sickle cell disease have increased susceptibility to infection caused by encapsulated organisms due to functional hyposplenism, and this is a common cause of death for this population [6,7]. It has been noted that when patients with sickle cell disease contract COVID-19, they have a higher risk of developing pneumonia and pain in comparison with patients who do not have this diagnosis [8]. In addition, sickle cell disease patients are more likely to be hospitalized when they contract COVID-19 [8].

According to the Centers for Disease Control and Prevention (CDC), there is a possibility that patients with sickle cell disease may be at higher risk of developing severe complications from COVID-19 [9]. In view of this, it would be expected that patients with sickle cell disease would benefit from administration of the COVID-19 vaccine. However, there is a relatively high rate of vaccine hesitancy in this patient population, which is due to a perceived lack of benefit from vaccination, and also lack of trust for the vaccine [10].

Patients with COVID-19 have a higher risk of developing thrombosis, as do patients with sickle cell disease [11,12]. This has led to concern that patients with sickle cell disease who acquire COVID-19 might be at substantial risk for developing complications due to venous thromboembolism. 

In view of the substantial short- and long-term morbidity associated with sickle cell disease, as well as vaccine hesitancy and the baseline increased risk of venous thromboembolism in this population, it appears reasonable to anticipate that these patients would be at risk for severe complications and poor clinical outcomes following SARS-CoV-2 infection; however, this has not been consistently observed [8,13-15].

## Materials and methods

This retrospective chart review included patients admitted to a large hospital system (which includes four hospitals) in the southeastern United States between March 2020 and December 2021 who had sickle cell disease as one of their discharge diagnoses. Patients with other infections were not excluded from this study, and no minimum length of hospitalization was required for inclusion in the study. Patients with incomplete medical records were not formally excluded from the study; however, each patient’s chart was manually reviewed and no incomplete medical records were identified. All patients were tested for COVID-19.

A discharge diagnosis of COVID-19 was also used to select patients for this study. The diagnosis of COVID-19 was indicated by the presence of a positive COVID-19 test result at any time during their admission. The specific test utilized was the Cepheid GeneXpert polymerase chain reaction test (Cepheid, Sunnyvale, CA, USA).

In this study data were compared between two patient groups: Group 1 had sickle cell disease without COVID-19, and it originally included 2687 patients. Group 2 had sickle cell disease plus COVID-19 as one of their discharge diagnoses, and it originally included 138 patients. Before data analysis was started for this study, randomized samples were obtained from both Group 1 and 2, so that 95 and 73 patients respectively were included from Group 1 and Group 2. 

Data points included patient sex, age group, genotype, hydroxyurea use, use of opioid analgesics for control of chronic pain, receipt of a COVID-19 vaccine prior to admission, and medical co-morbidities. Clinical outcomes that were evaluated in this study include requirement for blood transfusion during hospital admission, hospital length of stay, need for intensive care unit (ICU)-level care, 30-day readmissions, and mortality. Initial laboratory values (i.e., hemoglobin, hematocrit, serum creatinine, total bilirubin level, lactate dehydrogenase [LDH], and D-dimer) were also assessed for patients in both groups. Statistical significance (at p < 0.05) was assessed with Wilcoxon rank sum tests, and the analysis was powered by software R 4.3.1 (R Core Team, 2023, R Foundation for Statistical Computing, Vienna, Austria).

## Results

Patient demographics, medication use, and COVID-19 vaccination status 

Groups 1 and Group 2 were similar in terms of proportion of male patients (37% and 39%, respectively; p = 0.703), age distribution, genotype, use of hydroxyurea (43% and 48%, respectively; p = 0.537), use of opioid analgesics for chronic pain (77% and 73%, respectively; p = 0.529), and receipt of the COVID vaccine prior to admission (8% and 8%, respectively; p = 0.963); see Table 1.

**Table 1:** Demographic information and COVID-19 vaccination status for sickle cell disease patients

	Patients not diagnosed with COVID-19 during admission, N (%)	Patients diagnosed with COVID-19 during admission, N (%)	p-value
Sex			0.703
Male	35 (37%)	29 (39%)	
Female	60 (63%)	44 (60%)	
Age Group (in years)			0.379
18–24	22 (23%)	22 (30%)	
25–34	44 (46%)	28 (38%)	
35–44	19 (20%)	9 (12%)	
45–54	5 (5%)	8 (11%)	
55–64	3 (3%)	4 (5%)	
≥ 65	2 (2%)	2 (3%)	
Genotype			0.035
HbSS	58 (61%)	47 (64%)	
HbSC	28 (29%)	22 (30%)	
HbSβ+	8 (8%)	4 (5%)	
HbSβ0	1 (1%)	0 (0%)	
Patients taking hydroxyurea	41 (43%)	35 (48%)	0.537
Patients taking opioid analgesics for chronic pain prior to admission	73 (77%)	53 (73%)	0.529
Received COVID-19 vaccine prior to admission	8 (8%)	6 (8%)	0.963

Patient comorbidities

Groups 1 and 2 were also similar in regard to comorbidities including hypertension (13% and 14%, respectively; p = 0.839), diabetes (3% and 7%, respectively; p = 0.293), chronic kidney disease (12% and 11%, respectively; p = 0.928), pulmonary embolus/deep vein thrombosis (32% and 23%, respectively; p = 0.235), and acute chest syndrome (28% and 19%, respectively; p = 0.353); see Table 2. 

**Table 2:** Co-morbidities in sickle cell disease patients Note: both current and remote diagnoses are included.

	Patients not diagnosed with COVID-19 during admission, N (%)	Patients diagnosed with COVID-19 during admission, N (%)	p-value
Hypertension	12 (13%)	10 (14%)	0.839
Diabetes mellitus	3 (3%)	5 (7%)	0.296
Chronic kidney disease	11 (12%)	8 (11%)	0.928
Pulmonary embolus/deep vein thrombosis	30 (32%)	17 (23%)	0.235
Acute chest syndrome	27 (28%)	14 (19%)	0.353

Reasons for hospital admission

For Group 1 patients the most common reason for their emergency room visit was sickle cell vaso-occlusive crisis (76%). Vaso-occlusive disease was responsible for 86% of hospital admissions for Group 1 patients; see Table 3. For Group 2 patients 40% presented to the emergency room due to sickle cell vaso-occlusive crisis only, 7% had COVID-19 signs and symptoms only, and 52% had signs and symptoms of both COVID-19 and sickle cell vaso-occlusive crisis (Table 3). For 73% of Group 2 patients, the reason for hospital admission was both COVID-19 and sickle cell vaso-occlusive crisis. Sickle cell vaso-occlusive crisis was the reason for hospital admission for 26% of Group 2 patients. One percent of Group 2 patients were admitted due to COVID-19 only (that is, no symptoms of vaso-occlusive crisis).

**Table 3:** Reasons for presentation to emergency room and hospital admission for sickle cell disease patients

	Patients not diagnosed with COVID-19 during admission, N (%)	Patients diagnosed with COVID-19 during admission, N (%)	p-value
Reason for emergency room visit			<0.001
COVID-19 symptoms and signs only	0 (0%)	5 (7%)	
Sickle cell vaso-occlusive crisis only	72 (76%)	29 (40%)	
Signs and symptoms of both COVID-19 and sickle cell vaso-occlusive crisis	11 (12%)	38 (52%)	
Other	12 (13%)	1 (1%)	
Reason for hospital admission			<0.001
COVID-19	0 (0%)	1 (1%)	
Sickle cell vaso-occlusive crisis	82 (86%)	19 (26%)	
Both COVID-19 and sickle cell vaso-occlusive crisis	0 (0%)	53 (73%)	
Other	13 (14%)	0 (0%)	

Differences between groups on need for blood transfusions, hospital length of stay, need for ICU level care, 30-day readmissions, and mortality

Table 4 summarizes the impact of COVID-19 on the need for blood transfusions, hospital length of stay, need for ICU level care, 30-day readmissions, and mortality in Group 1 and Group 2 patients. In regard to the need for blood transfusions, for Group 1 patients, 26% required a simple blood transfusion, 9% required an exchange transfusion, and 8% required both simple and exchange transfusions during their hospital course. For Group 2 patients, 30% required a simple blood transfusion, 16% required an exchange transfusion, and none required a combination of both simple and exchange transfusions during their admission. 

**Table 4:** Impact of COVID-19 on need for blood transfusions, hospital length of stay, need for ICU level care, 30-day readmissions, and mortality

	Patients not diagnosed with COVID-19 during admission, N (%) unless otherwise noted	Patients diagnosed with COVID-19 during admission, N (%) unless otherwise noted	p-value
Required blood transfusion			0.034
Simple	25 (26%)	22 (30%)	
Exchange	9 (9%)	12 (16%)	
Both simple and exchange	8 (8%)	0 (0%)	
Length of stay (mean, in days)	7.05	7.64	0.985
Number of patients who received ICU care	6 (6.3%)	4 (5.5%)	1.000
Length of ICU stay (mean, in days)	3.17	6.25	0.863
Death	1 (1.05%)	1 (1.4%)	1.000
Number of readmissions in ≤30 days	25 (26.3%)	18 (24.7%)	0.807

Group 1 and Group 2 patients had hospital length of stays of 7.05 and 7.64 days, respectively (p = 0.985) (Table 4, above). ICU-level care was required for six patients (6.3%) in Group 1 and four patients (5.5%) in Group 2 (p = 1.00). The mean number of days in the ICU was 3.17 for Group 1 patients and 6.25 for Group 2 patients (p = 0.863). Readmissions within 30 days occurred for 25 patients (26.3%) in Group 1 and 18 patients (24.7%) in Group 2 (p = 0.807). Death occurred in one patient (1.05%) in Group 1, and one patient (1.4%) in Group 2 (p = 1.000); see Table 4, above.

Initial laboratory values among patients with and without COVID-19

Several laboratory values (total bilirubin, hemoglobin, hematocrit, creatinine, lactate dehydrogenase, and D-dimer) were evaluated to determine if they varied between Group 1 and Group 2 on the day of hospital admission (Table 5). No significant differences between Group 1 and Group 2 for any of these laboratory values were noted. 

**Table 5:** Initial laboratory values among sickle cell disease patients

	Patients not diagnosed with COVID-19 during admission	Patients diagnosed with COVID-19 during admission	p-value
Total bilirubin level (mean), mg/dL	3.8	3.1	0.56
Hemoglobin (mean), g/dL	8.6	8.6	0.74
Hematocrit (mean), %	25.1	25.1	0.82
Creatinine (mean), mg/dL	1.21	1.11	0.12
Lactate dehydrogenase (mean), U/L	388	466	0.51
D-dimer (mean), µg/l	4313	6577	0.32

Number of patients admitted, March 2020 through December 2021

**Figure 1:**
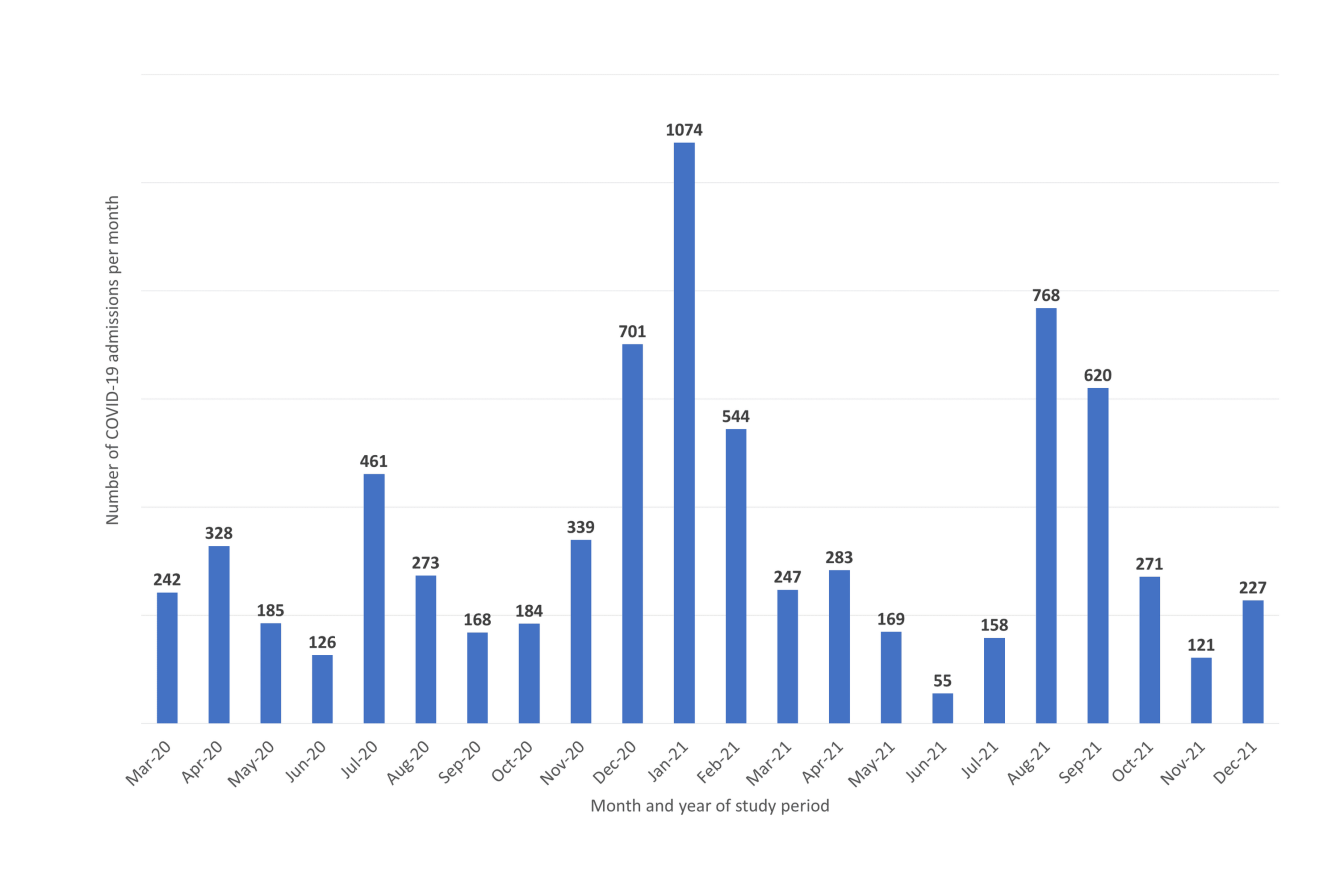
Numbers of patients admitted with COVID-19 during each month of study period

Figure 1 indicates the total number of patients (including those without sickle cell disease) who were admitted due to COVID-19 during each month of the study period.

## Discussion

Previous studies have found conflicting evidence regarding the impact of COVID-19 on sickle cell disease patients. Some of the literature has suggested that they may have worse clinical outcomes in comparison with the general population, while other publications have not reported this finding. Singh et al. observed higher rates of hospitalization, pneumonia, and pain among sickle cell disease patients with COVID-19 than among similar COVID-19 patients without sickle cell disease, but they found no differences in 14- or 30-day mortality [8]. Minniti et al. noted a case fatality rate of approximately 10% for sickle cell disease patients who acquired COVID-19 [16]. This compared with a case fatality rate of about 3% in the general population [16]. Payne et al. found that sickle cell disease-related death rates increased by about 12% in 2020, and about 8.4% of sickle cell disease deaths were attributed to COVID-19 that year [17]. A systemic review and meta-analysis by Borberema et al. that included 72 studies and 6011 sickle cell disease patients with COVID-19 reported a 3% case fatality rate [18]. Furthermore, 10% of these patients required care in the ICU, and mechanical ventilation was required for 4% of these patients [18].

At their usual baseline, patients with sickle cell disease are at increased risk for developing venous thromboembolism [12]. Since it has been observed that severe COVID-19 increases the risk of developing thromboembolism, initially there was concern that sickle patients who acquire this infection would be at particularly high risk of experiencing complications due to venous thromboembolic disease. However, Singh et al. noted that COVID-19 in sickle cell disease patients was not associated with a further increased risk of venous thromboembolism [19].

In their study of 1045 sickle cell disease patients (590 children and 455 adults) with COVID-19, Mucalo et al. found that the majority of them (62.2% of the children and 55.6% of the adults) had mild symptoms during their course of illness [20]. Abdulrahman et al. conducted a study of 1792 patients with COVID-19 and concluded that sickle cell disease is not a risk factor for severe outcomes due to COVID-19, since a similar rate of death was observed in patients with and without sickle cell disease [21]. They also noted that the use of noninvasive ventilation, high-flow nasal cannula, and mechanical ventilation was similar in both groups [21].

In this study, we sought to clarify whether hospitalized sickle cell disease patients with COVID-19 had significantly different clinical outcomes compared with those who did not have COVID-19. Given the high prevalence of chronic complications of sickle cell disease, which include a baseline hypercoagulable state, we hypothesized that the presence of COVID-19 would result in the following: (i) longer hospital length of stay, (ii) increased need for ICU-level care, (iii) higher rate of 30-day readmissions, and (iv) increased mortality rate. We also anticipated that COVID-19 might have a significant impact on some commonly measured laboratory values (including serum creatinine, hemoglobin, D-dimer, fibrinogen, and total bilirubin) in sickle cell disease patients. 

We instead found that there were virtually no significant differences in clinical outcomes between sickle cell disease patients who did and did not have COVID-19. Hoogenboom et al. have proposed one potential explanation for this surprising finding: that sickle cell disease-modifying therapies (e.g., hydroxyurea and transfusion) may also ameliorate some impacts of the virus [15]. While this is an intriguing hypothesis, Hoogenboom et al. acknowledged that prospective evaluations are needed to clarify the impact of sickle cell disease-modifying therapies on the clinical outcome of COVID-19 [15]. In our study we noted that both the frequency of blood transfusions and the use of hydroxyurea were similar in sickle cell disease patients who did and did not acquire COVID-19.

In addition, utilization of the COVID-19 vaccine, which was first released in December 2020, may have led to improvements in morbidity and mortality in sickle cell disease patients. A study by Han et al. looked at vaccination rates in patients with sickle cell disease at the University of Illinois and found that although vaccination rates were still lower for this patient population compared with the general population, about 50% received the first dose of the two-part COVID-19 vaccine series [22]. In our study, the same low rate of COVID-19 vaccination, that is, 8% was observed in sickle cell disease patients who did and did not acquire COVID-19.

Our study adds to the existing literature that suggests that the severity of COVID-19 is not worse for patients with sickle cell disease compared with the general population. Our findings confirm that COVID-19 has no impact on hospital length of stay, 30-day readmission rate, need for ICU-level care, or mortality for patients with sickle cell disease. This study also demonstrates that common laboratory values (i.e. total bilirubin, hemoglobin, hematocrit, creatinine, lactate dehydrogenase, and D-dimer) are not affected by COVID-19 in sickle cell disease patients.

The major limitation of our study is its relatively small size, that is, 197 patients (which were randomly selected from a total of 2825 patients). Also, although this study was carried out at four hospitals that are part of a large health system in the southeastern United States, it was not carried out as part of a large multicenter data review. For this reason, our results may be more difficult to generalize.

## Conclusions

Since the early stages of the COVID-19 pandemic, there has been concern that sickle cell disease patients may have worse clinical outcomes compared with the general population. However, the results of this study indicate that COVID-19 does not affect the clinical outcome of sickle cell disease patients in regard to hospital length of stay, requirement for ICU-level care, 30-day readmission rate, commonly ordered laboratory values, or mortality rate.
